# Activities of Eravacycline, Tedizolid, Norvancomycin, Nemonoxacin, Ceftaroline, and Comparators against 1,871 *Staphylococcus* and 1,068 *Enterococcus* Species Isolates from China: Updated Report of the CHINET Study 2019

**DOI:** 10.1128/spectrum.01715-22

**Published:** 2022-11-03

**Authors:** Li Ding, Yang Yang, Changhe Zheng, Gang Sun, Renru Han, Yan Guo, Dandan Yin, Shi Wu, Demei Zhu, Fupin Hu

**Affiliations:** a Institute of Antibiotics, Huashan Hospital, Fudan University, Shanghai, China; b Key Laboratory of Clinical Pharmacology of Antibiotics, Ministry of Health, Shanghai, China; c Yancheng Tinghu District People’s Hospital, Jiangsu, China; d The First Division Hospital of Xinjiang Corps, Xinjiang, China; Peking Union Medical College Hospital; the First Affiliated Hospital of Xinjiang Medical University; West China Hospital, Sichuan University; Children’s Hospital of Fudan University; the First Affiliated Hospital of Anhui Medical University; Tongji Hospital, Tongji Medical College, Huazhong University of Science & Technology; Ruijin Hospital, Shanghai Jiaotong University School of Medicine; the First Affiliated Hospital of China Medical University; Tianjin Medical University General Hospital; Sir Run Run Shaw Hospital, Zhejiang University School of Medicine; the First Affiliated Hospital of Kunming Medical University; the First Affiliated Hospital of Inner Mongolia Medical University; Gansu Provincial Hospital; Children’s Hospital of Shanghai; Beijing Hospital; the First Affiliated Hospital of Guangzhou Medical University; Pudong New Area People’s Hospital; Zhejiang Ningbo Zhenhai Longsai Hospital; the People’s Hospital of Zigui, Hubei Province; the First Affiliated Hospital of Harbin Medical University; Xiangya Hospital, Central South University; Shandong Provincial Hospital; Jinjiang Municipal Hospital; China-Japan Union Hospital, Jilin University; Sichuan Provincial People’s Hospital; Henan Provincial People’s Hospital; Shaanxi Provincial People’s Hospital; Jiangxi Provincial Children’s Hospital; Children’s Hospital of Shanxi; Nanjing Drum Tower Hospital, Affiliated Hospital of Nanjing; General Hospital of Ningxia Medical University; Shenzhen People’s Hospital; the Second Hospital of Hebei Medical University; Jinchang Hospital of Integrated Traditional Chinese and Western Medicine; the People’s Hospital of Ganxian; Guangrao County People’s Hospital; the People’s Hospital of Huixian, Henan Province; Central Hospital of Yingkou Development Zone, Liaoning Province; Huzhu County People’s Hospital, Qinghai Province; the People’s Hospital of Linshui, Sichuan Province; Lixin County People’s Hospital; Jiutai People's Hospital; Quanzhou First Hospital, Fujian; The First Affiliated Hospital of Xiamen University; Guizhou Provincial People’s Hospital; Shenzhen Children’s Hospital; Brown University

**Keywords:** *Staphylococcus* spp., *Enterococcus* spp., eravacycline, tedizolid, nemonoxacin, norvancomycin, antimicrobial susceptibility testing, MIC

## Abstract

To evaluate the *in vitro* activities of eravacycline, tedizolid, nemonoxacin, norvancomycin, and ceftaroline against Staphylococcus and *Enterococcus* species isolates were collected as part of the China Antimicrobial Surveillance Network (CHINET) in 2019 to provide susceptibility data for Staphylococcus spp. and *Enterococcus* spp. for their future development and application in clinical practice. Antimicrobial susceptibility testing was performed using the CLSI broth microdilution reference method. Eravacycline was highly active against Staphylococcus and *Enterococcus* species isolates, proved by the MIC_50/90_: 0.06/0.125, 0.06/0.25, 0.06/0.25, 0.06/0.25, 0.125/0.5, 0.125/0.25, and 0.03/0.06 mg/L for Staphylococcus aureus, methicillin-resistant S. aureus (MRSA), S. epidermidis, S. hominis, S. haemolyticus, Enterococcus faecalis, and E. faecium, respectively. S. aureus isolates tested were fully susceptible to tedizolid. Still, nonsusceptible isolates were found for E. faecalis (72/567 [12.7%]) and E. faecium (12/501 [2.4%]). Norvancomycin at 2 mg/L could inhibit 100% of Staphylococcus spp., while 1 mg/L of ceftaroline could inhibit 78.9% of MRSA and 99.9% of methicillin-susceptible S. aureus (MSSA) isolates. Additionally, nemonoxacin was also active against Staphylococcus and *Enterococcus* species isolates tested (shown by the following MIC_90_s and ranges, in milligrams per liter: 2 and ≤0.015 to 8 for MRSA, 0.25 and ≤0.015 to 4 for MSSA, 0.5 and ≤0.015 to 8 for S. epidermidis, and 4 and ≤0.015 to >32 for E. faecalis). In conclusion, both eravacycline and tedizolid were highly active against clinical isolates of Staphylococcus spp. and *Enterococcus* spp. recently collected across China. Nemonoxacin showed potent activity against Staphylococcus spp. and E. faecalis but limited activity against E. faecium. Norvancomycin and ceftaroline displayed highly potent activity against Staphylococcus spp.

**IMPORTANCE** Antimicrobial resistance has become a severe threat to global public health. According to statistics, nearly 700,000 people die from bacterial infections worldwide (J. O’Neill, *Antimicrobial Resistance: Tackling a Crisis for the Health and Wealth of Nations*, 2014; C. Y. Chin, K. A. Tipton, M. Farokhyfar, E. M. Burd, et al., Nat Microbiol 3:563–569, 2018, https://doi.org/10.1038/s41564-018-0151-5). The number of bacterial infections is expected to climb to 10 million by 2050, showing that bacterial resistance has become a significant problem that cannot be ignored. It is crucial to develop new antimicrobial agents to combat antimicrobial-resistant bacteria. In this study, we evaluated the *in vitro* activities of eravacycline, tedizolid, nemonoxacin, norvancomycin, and ceftaroline against Staphylococcus spp. and *Enterococcus* species isolates which were collected as part of CHINET in 2019. We believe that this study can provide susceptibility data for Staphylococcus spp. and *Enterococcus* spp. for their future development and application in clinical practice.

## INTRODUCTION

Antimicrobial resistance has severely threatened global public health (1). According to statistics, nearly 700,000 people die from bacterial infections worldwide (2–4). The number of bacterial infections is expected to climb to 10 million by 2050, showing that bacterial resistance has become a significant problem that cannot be ignored (5). The WHO published a list of bacteria urgently needing new antimicrobial agents in 2017. Carbapenem-resistant Acinetobacter baumannii, carbapenem-resistant Pseudomonas aeruginosa, carbapenem-resistant *Enterobacterales*, and extended-spectrum β-lactamase (ESBL)-producing *Enterobacterales* were present in the list of priority pathogens. In contrast, vancomycin-resistant enterococci (VRE) and methicillin-resistant Staphylococcus aureus (MRSA) were listed as high-priority pathogens (6).

S. aureus and *Enterococcus* spp. are common pathogens responsible for hospital- and community-acquired infections and can cause severe infections in health care facilities (7). S. aureus is one of the most common Gram-positive cocci that can cause community- and hospital-acquired pneumonia, skin and soft tissue infections, infective endocarditis, and bloodstream infections (8). MRSA is resistant to standard antimicrobial agents because it carries multiple drug resistance genes and virulence factors, increasing the mortality rate (9). The China Antimicrobial Surveillance Network (CHINET; www.chinets.com) of 2021 showed that more than 30% of S. aureus and 80% of Staphylococcus epidermidis isolates were MRSA and methicillin-resistant S. epidermidis (MRSE), respectively. *Enterococcus* spp., the second most common Gram-positive cocci for hospital and community infections, can cause bloodstream infections, infective endocarditis, and urinary tract infections. Moreover, enterococci are naturally resistant to cephalosporins, so the choice of antimicrobial agents for *Enterococcus* infections is minimal (10).

This study aimed to evaluate the *in vitro* activities of eravacycline, tedizolid, nemonoxacin, norvancomycin, and ceftaroline against Staphylococcus and *Enterococcus* species isolates collected as part of CHINET in 2019 to provide susceptibility data for Staphylococcus spp. and *Enterococcus* spp. for their future development and application in clinical practice.

## RESULTS

### Susceptibility of Staphylococcus spp.

The *in vitro* activities of norvancomycin, tedizolid, eravacycline, nemonoxacin, ceftaroline, and other comparator agents against 2,939 clinical isolates are summarized in Tables 1 and 2. Norvancomycin (MIC_50/90_, 0.5/1 mg/L; MIC range, 0.25 to 1 mg/L) and tedizolid (MIC_50/90_, 0.25/0.5 mg/L; MIC range, ≤0.06 to 0.5 mg/L) showed potent activities against S. aureus (*n* = 1,631). One hundred percent of the S. aureus strains were inhibited at the norvancomycin-susceptible MIC breakpoint (≤1 mg/L) and tedizolid-susceptible MIC breakpoint (≤0.5 mg/L) irrespective of whether the isolates were MRSA or methicillin-susceptible S. aureus (MSSA), similar to the case with vancomycin (100% susceptible) and linezolid (100% susceptible). The eravacycline MIC_90_ for S. aureus was 0.125 mg/L, with a 1-doubling-dilution shift being seen for MRSA. Similar to the case with tigecycline (98.2% susceptible), 84.5% and 97.1% of S. aureus isolates could be inhibited by 0.06 mg/L (FDA breakpoint) and 0.25 mg/L (EUCAST breakpoint) of eravacycline, respectively. In addition, ceftaroline could inhibit 92.9% of S. aureus isolates at 1 mg/L (Clinical and Laboratory Standards Institute [CLSI] ceftaroline-susceptible breakpoint), showing potent activity against MRSA (MIC_50/90_, 1/2 mg/L; MIC range, ≤0.25 to 8 mg/L) and MSSA (MIC_50/90_, 0.5/0.5 mg/L; MIC range, ≤0.25 to 2 mg/L). Nemonoxacin inhibited 94.6% of the S. aureus strains at 1 mg/L, showing potent activity against MRSA (MIC_50/90_, 0.06/2 mg/L; MIC range, ≤0.015 to 8 mg/L) and MSSA (MIC_50/90_, 0.03/0.25 mg/L; MIC range, ≤0.015 to 4 mg/L), better than levofloxacin (83.4% susceptible). Nitrofurantoin (99.6% susceptible) and trimethoprim-sulfamethoxazole (91.7% susceptible) also displayed potent activity against S. aureus. More than 60% of the S. aureus strains were susceptible to gentamicin (85% susceptible) and clindamycin (68.7% susceptible). Other comparator agents showed limited activity: erythromycin (32.7% susceptible) and penicillin (6.7% susceptible).

**TABLE 1:** *In vitro* activities of ceftaroline, eravacycline, tedizolid, norvancomycin, nemonoxacin, and comparative agents against Staphylococcus species isolates^*a*^

Organism(s) (no. of isolates)	Antimicrobial	Breakpoint(s)	MIC (mg/L)				% R	% S
			Range	50%	90%	Mode		
*S. aureus* (*n* = 1,631)	Eravacycline (FDA)	S ≤ 0.06	≤0.015 to 2	0.06	0.125	0.06	15.5^*b*^	84.5
	Eravacycline (EUCAST)	S ≤ 0.25, R > 0.25	≤0.015 to 2	0.06	0.125	0.06	2.9	97.1
	Tigecycline	S ≤ 0.5	≤0.06 to 2	0.125	0.25	0.125	1.8^*b*^	98.2
	Tedizolid	S ≤ 0.5, R ≥ 2	≤0.06 to 0.5	0.25	0.5	0.5	0	100
	Linezolid	S ≤ 4, R ≥ 8	0.25 to 4	2	4	2	0	100
	Norvancomycin	WT ≤ 1, NWT ≥ 2^*c*^	0.25 to 1	0.5	1	0.5	0^NWT^	100^WT^
	Vancomycin	S ≤ 2, R ≥ 16	0.25 to 2	1	1	1	0	100
	Nemonoxacin	S ≤ 1, R ≥ 2^*d*^	≤0.015 to 8	0.06	0.5	0.03	5.4	94.6
	Levofloxacin	S ≤ 1, R ≥ 4	≤0.25 to >32	≤0.25	8	≤0.25	16.1	83.4
	Ceftaroline	S ≤ 1, R ≥ 8	≤0.25 to 8	0.5	1	0.5	0.1	92.9
	Penicillin	S ≤ 0.125, R ≥ 0.25	≤0.06 to >8	>8	>8	>8	93.3	6.7
	Erythromycin	S ≤ 0.5, R ≥ 8	≤0.5 to >16	>16	>16	>16	61.3	32.7
	Clindamycin	S ≤ 0.5, R ≥ 4	≤0.5 to >16	≤0.5	>16	≤0.5	30.5	68.7
	Gentamicin	S ≤ 4, R ≥ 16	≤1 to >32	≤1	32	≤1	14.1	85
	Trimethoprim and sulfamethoxazole	S ≤ 2, R ≥ 4	≤0.25 to >8	≤0.25	2	≤0.25	8.3	91.7
	Nitrofurantoin	S ≤ 32, R ≥ 128	8 to 64	16	32	16	0	99.6
MRSA (*n* = 541)	Eravacycline (FDA)	S ≤ 0.06	≤0.015 to 2	0.06	0.25	0.06	24^*b*^	76
	Eravacycline (EUCAST)	S ≤ 0.25, R > 0.25	≤0.015 to 2	0.06	0.25	0.06	7.9	92.1
	Tigecycline	S ≤ 0.5	≤0.06 to 2	0.125	0.5	0.125	5.4^*b*^	94.6
	Tedizolid	S ≤ 0.5, R ≥ 2	≤0.06 to 0.5	0.25	0.5	0.25	0	100
	Linezolid	S ≤ 4, R ≥ 8	0.5 to 4	2	4	2	0	100
	Norvancomycin	WT ≤ 1, NWT ≥ 2^*c*^	0.5 to 1	0.5	1	0.5	0^NWT^	100^WT^
	Vancomycin	S ≤ 2, R ≥ 16	0.5 to 2	1	1	1	0	100
	Nemonoxacin	S ≤ 1, R ≥ 2^*d*^	≤0.015 to 8	0.06	2	0.03	13.7	86.3
	Levofloxacin	S ≤ 1, R ≥ 4	≤0.25 to >32	0.5	>32	≤0.25	28.7	71
	Ceftaroline	S ≤ 1, R ≥ 8	≤0.25 to 8	1	2	1	0.2	78.9
	Penicillin	S ≤ 0.125, R ≥ 0.25	0.25 to >8	>8	>8	>8	100	0
	Erythromycin	S ≤ 0.5, R ≥ 8	≤0.5 to >16	>16	>16	>16	79.1	16.5
	Clindamycin	S ≤ 0.5, R ≥ 4	≤0.5 to >16	>16	>16	>16	56	43.3
	Gentamicin	S ≤ 4, R ≥ 16	≤1 to >32	≤1	>32	≤1	22.2	76.5
	Trimethoprim and sulfamethoxazole	S ≤ 2, R ≥ 4	≤0.25 to >8	≤0.25	0.5	≤0.25	5.5	94.5
	Nitrofurantoin	S ≤ 32, R ≥ 128	8 to 64	16	32	16	0	99.3
MSSA (*n* = 1,090)	Eravacycline (FDA)	S ≤ 0.06	≤0.015 to 1	0.06	0.125	0.03	11.2^*b*^	88.8
	Eravacycline (EUCAST)	S ≤ 0.25, R > 0.25	≤0.015 to 1	0.06	0.125	0.03	0.5	99.5
	Tigecycline	S ≤ 0.5	≤0.06 to 1	0.125	0.25	0.125	0.1^*b*^	99.9
	Tedizolid	S ≤ 0.5, R ≥ 2	≤0.06 to 0.5	0.5	0.5	0.5	0	100
	Linezolid	S ≤ 4, R ≥ 8	0.25 to 4	2	4	2	0	100
	Norvancomycin	WT ≤ 1, NWT ≥ 2^*c*^	0.25 to 1	0.5	1	0.5	0^NWT^	100^WT^
	Vancomycin	S ≤ 2, R ≥ 16	0.25 to 2	1	1	1	0	100
	Nemonoxacin	S ≤ 1, R ≥ 2^*d*^	≤0.015 to 4	0.03	0.25	0.03	1.3	98.7
	Levofloxacin	S ≤ 1, R ≥ 4	≤0.25 to >32	≤0.25	2	≤0.25	9.8	89.5
	Ceftaroline	S ≤ 1, R ≥ 8	≤0.25 to 2	0.5	0.5	0.5	0	99.9
	Penicillin	S ≤ 0.125, R ≥ 0.25	≤0.06 to >8	8	>8	>8	89.9	10.1
	Erythromycin	S ≤ 0.5, R ≥ 8	≤0.5 to >16	>16	>16	>16	52.4	40.8
	Clindamycin	S ≤ 0.5, R ≥ 4	≤0.5 to >16	≤0.5	>16	≤0.5	17.8	81.4
	Gentamicin	S ≤ 4, R ≥ 16	≤1 to >32	≤1	16	≤1	10.1	89.3
	Trimethoprim and sulfamethoxazole	S ≤ 2, R ≥ 4	≤0.25 to >8	≤0.25	2	≤0.25	9.7	90.3
	Nitrofurantoin	S ≤ 32, R ≥ 128	8 to 64	16	32	16	0	99.7
S. epidermidis (*n* = 121)	Eravacycline	NA	≤0.015 to 1	0.06	0.25	0.03, 0.125	NA	NA
	Tigecycline	NA	≤0.06 to 0.5	0.125	0.25	0.125	NA	NA
	Tedizolid	NA	≤0.06 to 0.25	0.125	0.25	0.125	NA	NA
	Linezolid	S ≤ 4, R ≥ 8	0.5 to 2	1	2	1	0	100
	Norvancomycin	S ≤ 2, R ≥ 4	≤0.015 to 2	1	1	1	0	100
	Vancomycin	S ≤ 4, R ≥ 32	0.5 to 4	2	2	2	0	100
	Nemonoxacin	NA	≤0.015 to 8	0.06	0.5	0.03	NA	NA
	Levofloxacin	S ≤ 1, R ≥ 4	≤0.25 to >32	0.5	16	≤0.25	43.8	53.7
	Ceftaroline	NA	≤0.25 to 2	≤0.25	0.5	≤0.25	NA	NA
	Penicillin	S ≤ 0.125, R ≥ 0.25	≤0.06 to >8	8	>8	>8	95.9	4.1
	Erythromycin	S ≤ 0.5, R ≥ 8	≤0.5 to 32	>16	>16	>16	77.7	22.3
	Clindamycin	S ≤ 0.5, R ≥ 4	≤0.5 to >16	≤0.5	>16	≤0.5	28.9	68.6
	Gentamicin	S ≤ 4, R ≥ 16	≤1 to >32	≤1	32	≤1	20.7	71.1
	Trimethoprim and sulfamethoxazole	S ≤ 2, R ≥ 4	≤0.25 to >8	4	>8	≤0.25	57	43
	Nitrofurantoin	S ≤ 32, R ≥ 128	8 to 256	16	32	16	0.8	99.2
S. hominis (*n* = 61)	Eravacycline	NA	≤0.015 to 1	0.06	0.25	0.03	NA	NA
	Tigecycline	NA	≤0.06 to 0.5	0.125	0.25	≤0.06	NA	NA
	Tedizolid	NA	≤0.06 to 0.5	0.125	0.25	0.125	NA	NA
	Linezolid	S ≤ 4, R ≥ 8	0.5 to 8	1	2	1	1.6	98.4
	Norvancomycin	S ≤ 1, R ≥ 2	0.25 to 1	0.5	1	0.5	0	100
	Vancomycin	S ≤ 4, R ≥ 32	0.5 to 2	1	1	1	0	100
	Nemonoxacin	NA	≤0.015 to 8	0.5	4	0.5	NA	NA
	Levofloxacin	S ≤ 1, R ≥ 4	≤0.25 to >32	8	>32	>32	59	39.3
	Ceftaroline	NA	≤0.25 to 4	1	2	1	NA	NA
	Penicillin	S ≤ 0.125, R ≥ 0.25	≤0.06 to >8	8	>8	>8	91.8	8.2
	Erythromycin	S ≤ 0.5, R ≥8	≤0.5 to >16	>16	>16	>16	91.8	8.2
	Clindamycin	S ≤ 0.5, R ≥ 4	≤0.5 to >16	≤0.5	>16	≤0.5	37.7	62.3
	Gentamicin	S ≤ 4, R ≥ 16	≤1 to 32	2	8	≤1	4.9	83.6
	Trimethoprim and sulfamethoxazole	S ≤ 2, R ≥ 4	≤0.25 to >8	4	>8	4	65.6	34.4
	Nitrofurantoin	S ≤ 32, R ≥ 128	8 to 32	16	32	16	0	100
*S. haemolyticus* (*n* = 58)	Eravacycline	NA	≤0.015 to 2	0.125	0.5	0.25	NA	NA
	Tigecycline	NA	≤0.06 to 1	0.25	0.5	0.25	NA	NA
	Tedizolid	NA	0.125 to 0.5	0.125	0.25	0.125	NA	NA
	Linezolid	S ≤ 4, R ≥ 8	1 to 2	2	2	2	0	100
	Norvancomycin	S ≤ 2, R ≥ 4	0.5 to 2	1	2	1	0	100
	Vancomycin	S ≤ 4, R ≥ 32	0.5 to 4	1	2	1	0	100
	Nemonoxacin	NA	0.03 to 4	0.5	2	1	NA	NA
	Levofloxacin	S ≤ 1, R ≥ 4	≤0.25 to >32	8	32	8	74.1	25.9
	Ceftaroline	NA	≤0.25 to 8	2	4	2	NA	NA
	Penicillin	S ≤ 0.125, R ≥ 0.25	≤0.06 to >8	>8	>8	>8	91.4	8.6
	Erythromycin	S ≤ 0.5, R ≥ 8	≤0.5 to >16	>16	>16	>16	96.6	3.4
	Clindamycin	S ≤ 0.5, R ≥ 4	≤0.5 to >16	≤0.5	>16	≤0.5	37.9	60.3
	Gentamicin	S ≤ 4, R ≥ 16	≤1 to >32	16	>32	≤1	53.4	41.4
	Trimethoprim and sulfamethoxazole	S ≤ 2, R ≥ 4	≤0.25 to >8	1	>8	≤0.25, >8	32.8	67.2
	Nitrofurantoin	S ≤ 32, R ≥ 128	16 to >256	16	32	16	1.7	98.3

a R, resistant; S, susceptible; NWT, non-wild type; WT, wild type; NA, not available.

b Nonsusceptible rate for eravacycline and tigecycline.

c Epidemiological cutoff values for norvancomycin against Staphylococcus spp.

d Tentative clinical breakpoints of nemonoxacin for Staphylococcus aureus.

**TABLE 2:** *In vitro* activities of eravacycline, tedizolid, norvancomycin, nemonoxacin, and comparative agents against *Enterococcus* species isolates

Organism(s) (no. of isolates)	Antimicrobial	Breakpoint(s)	MIC (mg/L)				% R	% S
			Range	50%	90%	Mode		
E. faecalis (*n* = 567)	Eravacycline (FDA)	S ≤ 0.06	≤0.015 to 1	0.03	0.06	0.03	3^*a*^	97
	Eravacycline (EUCAST)	S ≤ 0.125, R > 0.125	≤0.015 to 1	0.03	0.06	0.03	0.5	99.5
	Tigecycline	S ≤ 0.25	≤0.06 to 2	0.125	0.25	0.125	1.4^*a*^	98.6
	Tedizolid	S ≤ 0.5	≤0.06 to >8	0.5	1	0.5	12.7^*a*^	87.3
	Linezolid	S ≤ 2, R ≥ 8	0.125 to >8	2	8	2	11.6	84.7
	Vancomycin	S ≤ 4, R ≥ 32	≤0.125 to 4	1	2	1	0	100
	Nemonoxacin	NA	≤0.015 to >32	0.25	4	0.25	NA	NA
	Levofloxacin	S ≤ 2, R ≥ 8	≤0.25 to >32	2	32	2	28.7	68.4
	Ceftaroline	NA	≤0.25 to >32	2	4	2	NA	NA
	Ampicillin	S ≤ 8, R ≥ 16	≤1 to >64	≤1	2	≤1	1.8	98.2
	Penicillin	S ≤ 8, R ≥ 16	0.5 to >8	2	4	2	4.4	95.6
	Gentamicin (high level)	S ≤ 500, R ≥ 1,000	≤500 to >500	≤500	>500	≤500	28.2	71.8
	Nitrofurantoin	S ≤ 32, R ≥ 128	4 to >256	16	16	16	0.7	98.8
E. faecium (*n* = 501)	Eravacycline (FDA)	S ≤ 0.06	≤0.015 to 2	0.03	0.125	0.03	11.8^*a*^	88.2
	Eravacycline (EUCAST)	S ≤ 0.125, R > 0.125	≤0.015 to 2	0.03	0.125	0.03	5.6	94.4
	Tigecycline	S ≤ 0.25, R > 0.25	≤0.06 to >4	≤0.06	0.125	≤0.06	3.2	96.8
	Tedizolid	S ≤ 0.5	≤0.06 to 8	0.5	0.5	0.5	2.4^*a*^	97.6
	Linezolid	S ≤ 2, R ≥ 8	0.5 to >8	2	2	2	1.8	93.4
	Vancomycin	S ≤ 4, R ≥ 32	0.25 to >8	1	2	1	0	94
	Norvancomycin	NA	0.125 to >32	0.5	2	0.5		
	Nemonoxacin	NA	≤0.015 to >32	8	32	8	NA	NA
	Levofloxacin	S ≤ 2, R ≥ 8	≤0.25 to >32	>32	>32	>32	84	8.4
	Ampicillin	S ≤ 8, R ≥ 16	≤1 to >64	>64	>64	>64	85.6	14.4
	Gentamicin (high level)	S ≤ 500, R ≥ 1,000	≤500 to >500	≤500	>500	≤500	48.7	51.3
	Nitrofurantoin	S ≤ 32, R ≥ 128	4 to 256	64	128	64	20.2	36.3
Vancomycin-resistant E. faecium (*n* = 30)	Eravacycline (FDA)	S ≤ 0.06	≤0.015 to 2	0.03	0.125	0.03	23.3^*a*^	76.7
	Eravacycline (EUCAST)	S ≤ 0.125, R > 0.125	≤0.015 to 2	0.03	0.125	0.03	10.0	90.0
	Tigecycline	S ≤ 0.25, R > 0.25	≤0.06 to >4	≤0.06	1	≤0.06	13.3	86.7
	Tedizolid	S ≤ 0.5	≤0.06 to 0.5	0.25	0.5	0.5	0^*a*^	100
	Linezolid	S ≤ 2, R ≥ 8	0.5 to 4	2	2	2	0	93.3
	Vancomycin	S ≤ 4, R ≥ 32	8 to >8	>8	>8	>8	0	0
	Norvancomycin	NA	8 to >32	>32	>32	>32		
	Nemonoxacin	NA	0.25 to 32	4	16	4	NA	NA
	Levofloxacin	S ≤ 2, R ≥ 8	1 to >32	>32	>32	>32	93.3	6.7
	Ampicillin	S ≤ 8, R ≥ 16	≤1 to >64	>64	>64	>64	93.3	6.7
	Gentamicin (high level)	S ≤ 500, R ≥ 1,000	≤500 to >500	>500	>500	>500	53.3	46.7
	Nitrofurantoin	S ≤ 32, R ≥ 128	16 to 128	64	128	64	23.3	33.3
Vancomycin-susceptible E. faecium (*n* = 471)	Eravacycline (FDA)	S ≤ 0.06	≤0.015 to 2	0.03	0.125	0.03	11^*a*^	89
	Eravacycline (EUCAST)	S ≤ 0.125, R > 0.125	≤0.015 to 2	0.03	0.125	0.03	5.3	94.7
	Tigecycline	S ≤ 0.25, R > 0.25	≤0.06 to >4	≤0.06	0.125	≤0.06	2.5	97.5
	Tedizolid	S ≤ 0.5	0.125 to 8	0.5	0.5	0.5	2.5^*a*^	97.5
	Linezolid	S ≤ 2, R ≥ 8	0.5 to >8	2	2	2	1.9	93.4
	Vancomycin	S ≤ 4, R ≥ 32	0.25 to 4	1	2	1	0	100
	Norvancomycin	NA	0.5	1	0.5			
	Nemonoxacin	NA	≤0.015 to >32	8	32	8	NA	NA
	Levofloxacin	S ≤ 2, R ≥ 8	≤0.25 to >32	>32	>32	>32	83.4	8.5
	Ampicillin	S ≤ 8, R ≥ 16	≤1 to >64	>64	>64	>64	85.1	14.9
	Gentamicin (high level)	S ≤ 500, R ≥ 1,000	≤500 to >500	≤500	>500	≤500	48.4	51.6
	Nitrofurantoin	S ≤ 32, R ≥ 128	4 to 256	64	128	64	20	36.5

a Nonsusceptible rate for eravacycline, tigecycline, and tedizolid.

Norvancomycin (MIC_50/90_, 1/1 mg/L; 100% susceptible) and tedizolid (MIC_50/90_, 0.125/0.25 mg/L; MIC range, ≤0.06 to 0.25 mg/L) showed great activity against S. epidermidis (*n* = 121), similar to vancomycin (100% susceptible, MIC_50/90_, 2/2 mg/L) and linezolid (100% susceptible; MIC_50/90_, 1/2 mg/L). Ceftaroline (MIC_50/90_, ≤0.25/0.5 mg/L; MIC range, ≤0.25 to 2 mg/L) showed potent *in vitro* activity against S. epidermidis. In addition, the MIC_50/90_ values of eravacycline against S. epidermidis were ≤0.06/0.25 mg/L, similar to those of tigecycline (MIC_50/90_, 0.125/0.25 mg/L). Nemonoxacin (MIC_50/90_, 0.06/0.5 mg/L; MIC range, ≤0.015 to 8 mg/L) was highly active against S. epidermidis, better than levofloxacin (83.4% susceptible; MIC_50/90_, 0.5/16 mg/L; MIC range, ≤0.25 to >32 mg/L). More than 60% of the S. epidermidis strains were susceptible to nitrofurantoin (99.2% susceptible), gentamicin (71.1% susceptible), and clindamycin (68.6% susceptible). Other comparator agents showed limited activity: erythromycin (22.3% susceptible), trimethoprim-sulfamethoxazole (43% susceptible), and penicillin (4.1% susceptible).

Norvancomycin (100% susceptible; MIC_50/90_, 0.5/1 mg/L) and tedizolid (MIC_50/90_, 0.125/0.25 mg/L; MIC range, ≤0.06 to 0.5 mg/L) also displayed potent *in vitro* activity against Staphylococcus hominis (*n* = 61), similar to vancomycin (100% susceptible; MIC_50/90_, 1/1 mg/L) and linezolid (98.4% susceptible; MIC_50/90_, 1/2 mg/L). Ceftaroline (MIC_50/90_, 1/2 mg/L; MIC range, ≤0.25 to 4 mg/L) showed potent *in vitro* activity against S. hominis. In addition, eravacycline (MIC_50/90_, 0.06/0.25 mg/L; MIC range, ≤0.015 to 1 mg/L) was highly active against S. hominis, similar to tigecycline (MIC_50/90_, 0.125/0.25 mg/L; MIC range, ≤0.06 to 0.5 mg/L). Nemonoxacin (MIC_50/90_, 0.5/4 mg/L; MIC range, ≤0.015 to 8 mg/L) was also highly active against S. hominis, better than levofloxacin (39.3% susceptible; MIC_50/90_, 8/>32 mg/L; MIC range, ≤0.25 to >32 mg/L). More than 60% of the S. hominis strains were susceptible to nitrofurantoin (100% susceptible), gentamicin (83.6% susceptible), and clindamycin (62.3% susceptible). Other comparator agents showed limited activity: erythromycin (8.2% susceptible), trimethoprim-sulfamethoxazole (34.4% susceptible), and penicillin (8.2% susceptible).

Norvancomycin (100% susceptible; MIC_50/90_, 1/2 mg/L) and tedizolid (MIC_50/90_, 0.125/0.25 mg/L; MIC range, 0.125 to 0.5 mg/L) also displayed potent activity against *S. haemolyticus* (*n* = 58), similar to vancomycin (100% susceptible; MIC_50/90_, 1/2 mg/L) and linezolid (100% susceptible; MIC_50/90_, 2/2 mg/L). The MIC_50/90_ and MIC range of ceftaroline against Staphylococcus haemolyticus were 2/4 mg/L and ≤0.25 to 8 mg/L, respectively. In addition, eravacycline (MIC_50/90_, 0.125/0.5 mg/L; MIC range, ≤0.015 to 2 mg/L) was highly active against *S. haemolyticus*, similar to tigecycline (MIC_50/90_, 0.25/0.5 mg/L; MIC range, ≤0.06 to 1 mg/L). Nemonoxacin (MIC_50/90_, 0.5/2 mg/L; MIC range, 0.03 to 4 mg/L) was highly active against S. hominis, better than levofloxacin (25.9% susceptible; MIC_50/90_, 8/32 mg/L). More than 60% of the *S. haemolyticus* strains were susceptible to nitrofurantoin (98.3% susceptible), trimethoprim-sulfamethoxazole (67.2% susceptible), and clindamycin (60.3% susceptible). Other comparator agents showed limited activity: erythromycin (3.4% susceptible), penicillin (8.6% susceptible), and gentamicin (41.4% susceptible).

### Susceptibility of *Enterococcus* spp.

Enterococcus faecalis strains were highly inhibited by tedizolid (MIC_50/90_, 0.5/1 mg/L; MIC range, ≤0.06 to >8 mg/L; 87.3% susceptible), similar to the case with linezolid (MIC_50/90_, 2/8 mg/L; MIC range, 0.125 to >8 mg/L; 84.7% susceptible). Eravacycline (MIC_50/90_, 0.03/0.06 mg/L; MIC range, ≤0.015 to 1 mg/L) also displayed potent activity against E. faecalis, evidenced by inhibition of 97% of E. faecalis isolates at 0.06 mg/L (FDA breakpoint) and 99.5% of E. faecium isolates at 0.125 mg/L (EUCAST breakpoint), similar to the case with tigecycline (98.6% susceptible; MIC_50/90_, 0.125/0.25 mg/L). More than 90% of the E. faecalis strains were susceptible to vancomycin (100% susceptible), ampicillin (98.2% susceptible), and nitrofurantoin (98.8% susceptible). More than 60% of the E. faecalis strains were susceptible to levofloxacin (68.4% susceptible) and gentamicin (high level; 71.8% susceptible). Additionally, nemonoxacin (MIC_50/90_, 0.25/4 mg/L; MIC range, ≤0.015 to >32mg/L) displayed high activity against E. faecalis, better than that of levofloxacin (MIC_50/90_, 2/32 mg/L; 68.4% susceptible).

Tedizolid (MIC_50/90_, 0. 5/0.5 mg/L; MIC range, ≤0.006 to 8 mg/L) could inhibit 97.6% of E. faecium isolates at 0.5 mg/L, similar to linezolid (93.4% susceptible). But the tedizolid MIC_90_ for E. faecium was 0.5 mg/L, regardless of its vancomycin susceptibility, 4-fold lower than that of linezolid (2 mg/L). Eravacycline (MIC_50/90_, 0.03/0.125 mg/L; MIC range, ≤0.015 to 2 mg/L) also showed potent activity against E. faecium, regardless of its vancomycin susceptibility, evidenced by inhibition of 88.2% of E. faecium isolates at 0.06 mg/L (FDA breakpoint) and 94.4% of E. faecium isolates at 0.125 mg/L (EUCAST breakpoint), similar to tigecycline (96.8% susceptible; MIC_50/90_, ≤0.06/0.125 mg/L). Additionally, norvancomycin (MIC_50/90_, 0.5/2 mg/L; MIC range, 0.125 to >32mg/L) also displayed potent activity against E. faecium, similar to vancomycin (94% susceptible; MIC_50/90_, 1/2 mg/L). Other comparator agents showed limited activity: levofloxacin (8.4% susceptible), gentamicin (high-level, 51.3% susceptible), nitrofurantoin (36.3% susceptible), and ampicillin (14.4% susceptible). Vancomycin-resistant E. faecium was more resistant to ampicillin, levofloxacin, gentamicin (high level), nitrofurantoin, and tigecycline than vancomycin-susceptible E. faecium. In contrast, vancomycin-susceptible E. faecium was more resistant to linezolid than vancomycin-resistant E. faecium.

## DISCUSSION

Antimicrobial resistance complicates the treatment of severe infections, causing increased morbidity, mortality, and additional costs, and contributes to a large proportion of the global antimicrobial resistance burden (1). A study by Gagliotti et al. showed that MRSA percentages among S. aureus bloodstream infections decreased from 30.2% in 2005 to 16.3% in 2018 in Europe (11). Similarly, results from the China Antimicrobial Surveillance Network (CHINET; www.chinets.com) also showed that the MRSA percentages decreased from 69.0% in 2005 to 30.5% in 2022. The reasons for this may be related to the effectiveness of medical institutions in recent years in actively implementing policies on the rational clinical application of antimicrobial agents and strengthening hospital infection control. With the standardized management and rational application of antimicrobial drug application in hospitals, the strengthening of laboratory and clinical communication ability, and the awareness of prevention and control of antibiotic-resistant bacterial infections, the epidemic spread of antibiotic-resistant bacteria has been curbed to some extent. Research by Brinkwirth et al. revealed that VRE proportions ranged between 0% and 40% among all *Enterococcus* species isolates from patients with hospital-acquired infections (HAI) in hospitals and intensive care units (ICUs) in the WHO European Region (12). According to data on E. faecium isolates from the Antibiotic Resistance Surveillance of the Robert Koch Institute, the proportion of existing vancomycin resistance in German hospitals increased from 11.2% in 2014 to 26.1% in 2017 (13, 14). The China Antimicrobial Surveillance Network (www.chinets.com) of 2021 showed that 1.4% of the E. faecium isolates were resistant to vancomycin. Therefore, developing new antimicrobial agents to combat antimicrobial-resistant bacteria is crucial.

**FIG 1:**
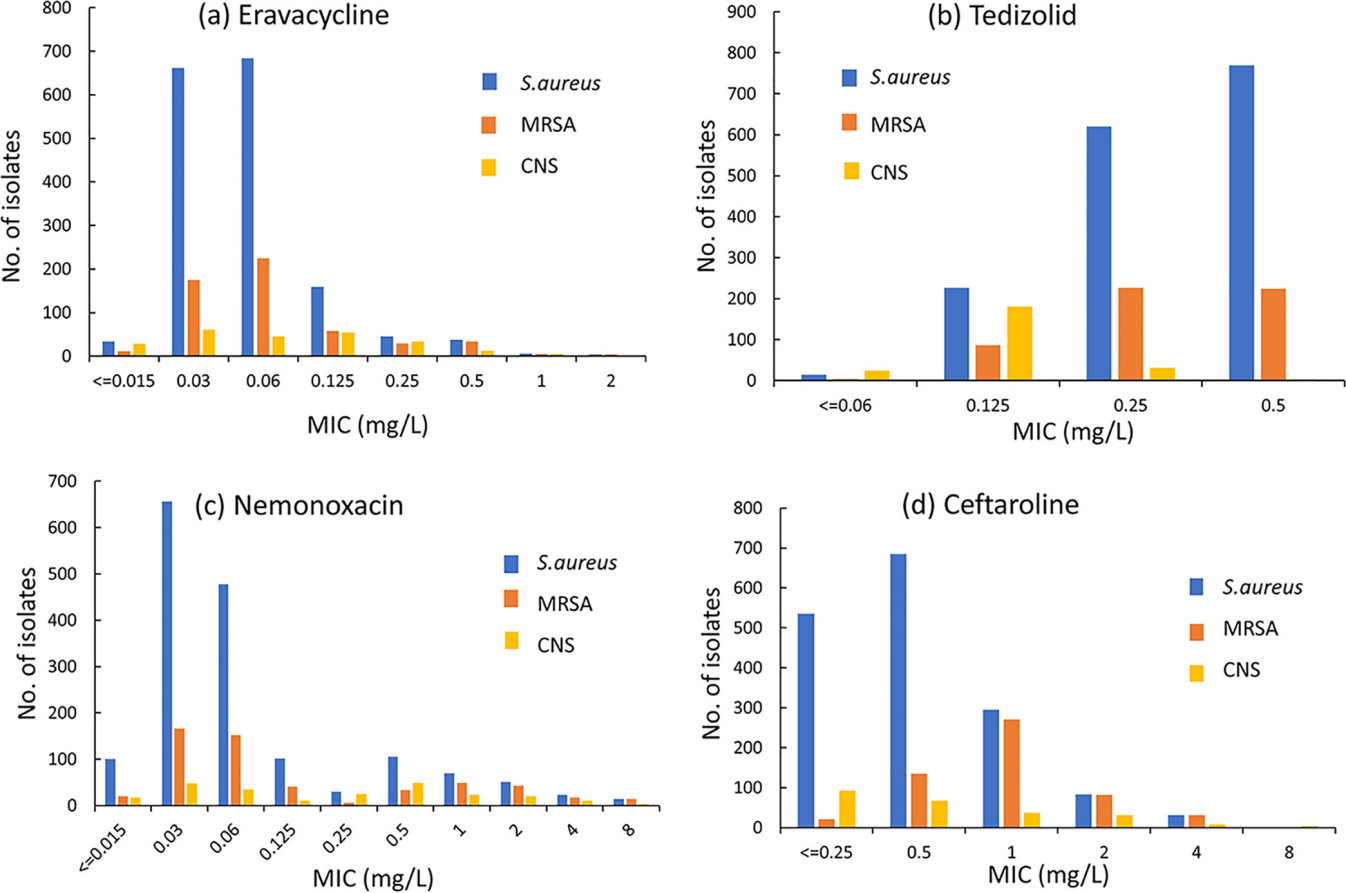
Distribution of MICs of eravacycline (a), tedizolid (b), nemonoxacin (c), and ceftaroline (d) against Staphylococcus spp. CNS, coagulase-negative staphylococci.

**FIG 2:**
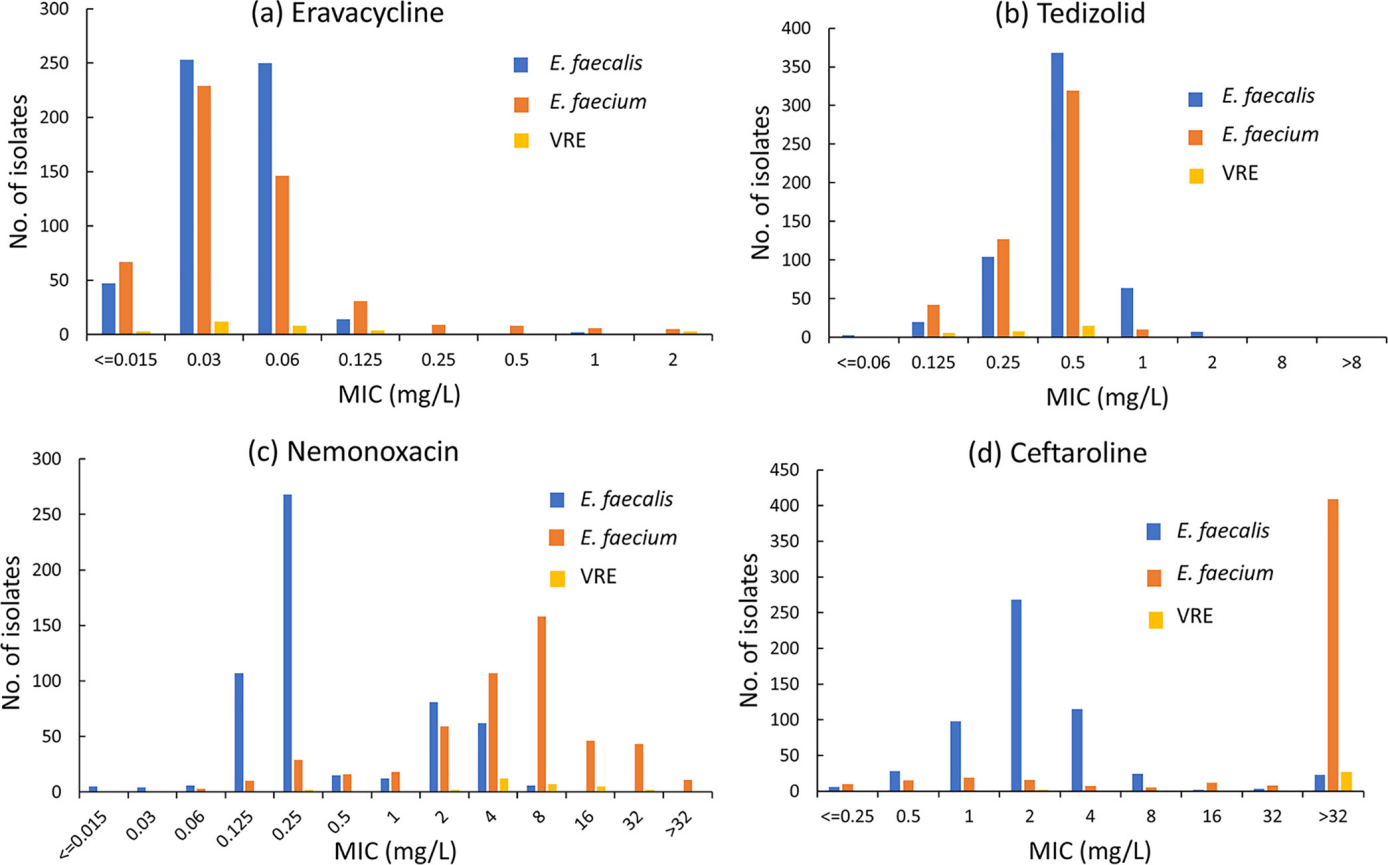
Distribution of MICs of eravacycline (a), tedizolid (b), nemonoxacin (c), and ceftaroline (d) against *Enterococcus* spp.

**FIG 3:**
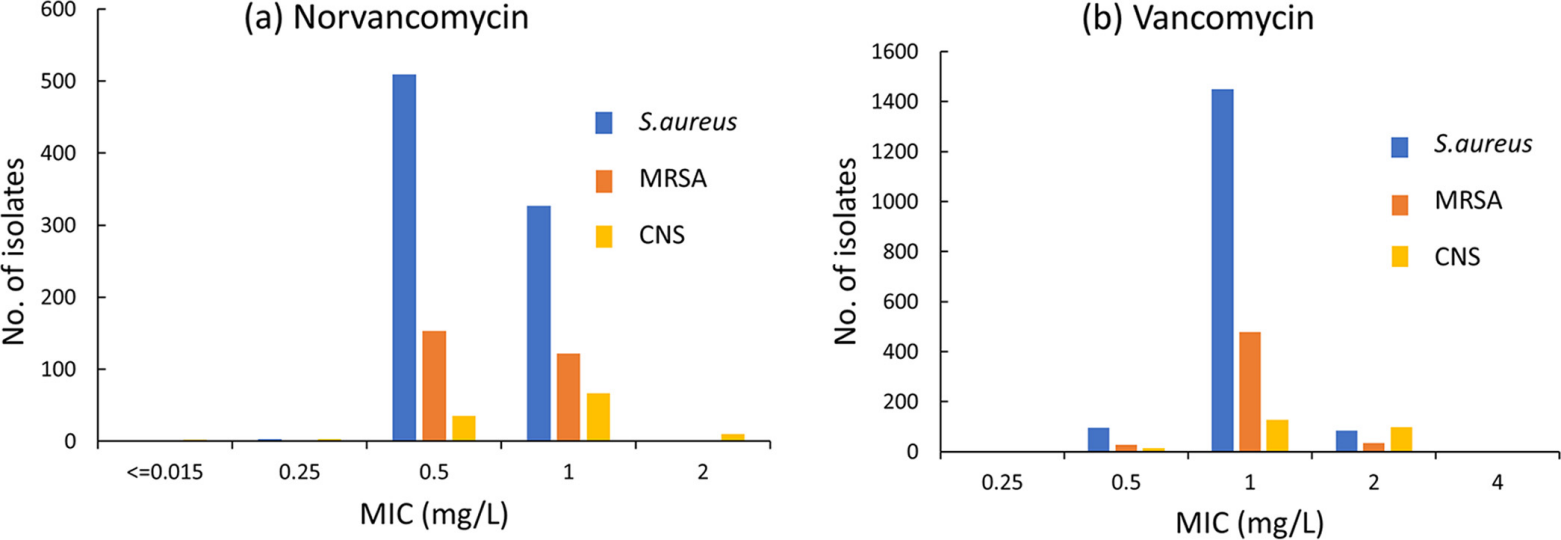
Distribution of MICs of norvancomycin (a) and vancomycin (b) against Staphylococcus spp.

**FIG 4:**
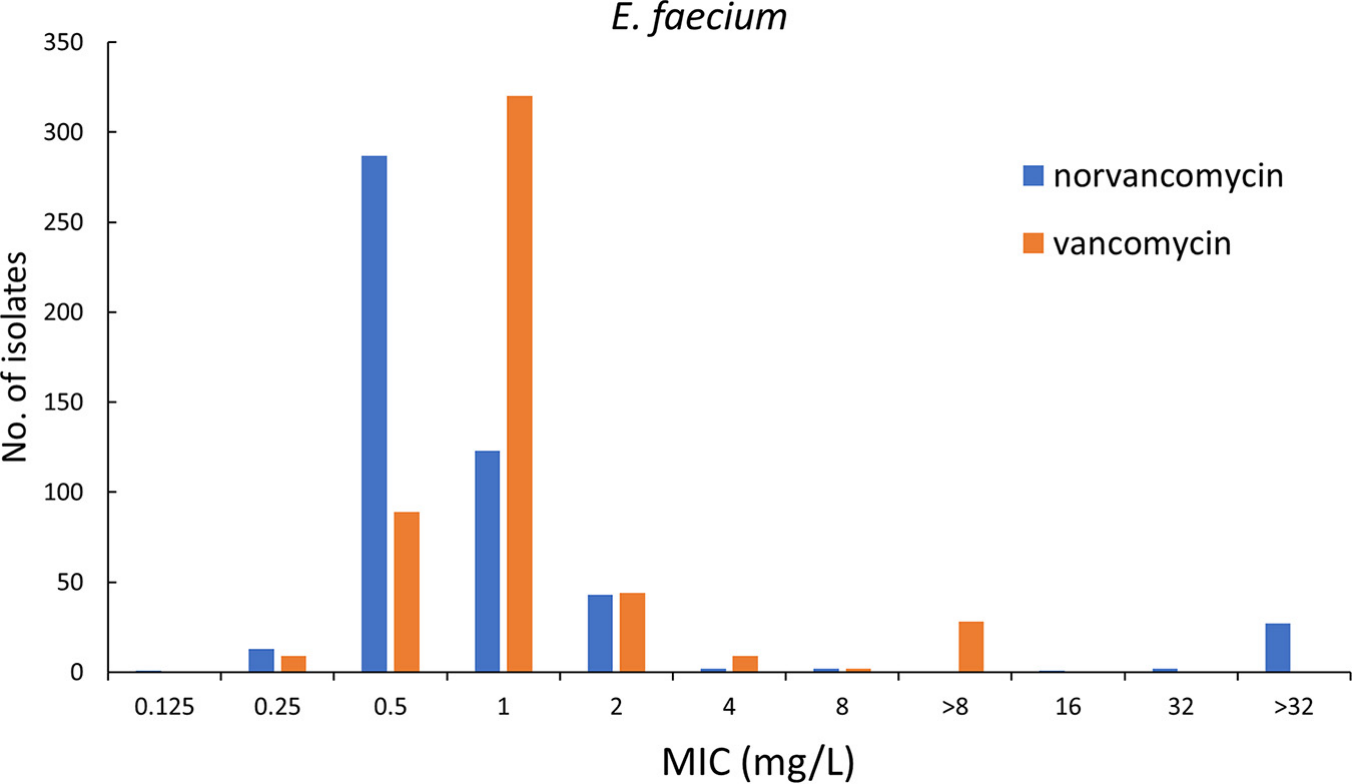
Distribution of MICs of norvancomycin and vancomycin against E. faecium.

Eravacycline is a fluorinated tetracycline similar in structure to tigecycline (15). Our study showed that the MIC_50/90_ values of eravacycline against S. aureus, MRSA, S. epidermidis, S. hominis, *S. haemolyticus*, E. faecalis, and E. faecium isolates were 0.06/0.125, 0.06/0.25, 0.06/0.25, 0.06/0.25, 0.125/0.5, 0.03/0.06, and 0.03/0.125 mg/L, respectively (Fig. 1 and 2). Eravacycline presented MIC values equal to or lower than those of tigecycline for most strains tested in this study. Similarly, Morrissey et al. (16) reported excellent *in vitro* activity for eravacycline against Gram-positive bacteria, including MRSA (MIC_50/90_, 0.06/0.12 mg/L), E. faecalis (MIC_50/90_, 0.06/0.06 mg/L), and E. faecium (MIC_50/90_, 0.03/0.06 mg/L). Tedizolid, an oxazolidinone, is a fully synthetic antibiotic that prevents bacterial protein synthesis by blocking the formation of a functional 70S initiation complex (17). Although structurally similar to linezolid, tedizolid achieved enhanced interactions at the binding site, thus increasing potency in some linezolid-resistant strains (18). Tedizolid demonstrated 4- to 8-fold-lower MIC_90_ values than linezolid for populations of Gram-positive pathogens in this study, including MRSA (0.5 mg/L versus 4 mg/L), E. faecalis (1 mg/L versus 8 mg/L), and E. faecium (0.5 mg/L versus 2 mg/L) (Fig. 1 and 2). Similarly, our previous *in vitro* studies comparing tedizolid and linezolid against staphylococci and enterococci collected from China in 2018 reported 4-fold improvements in the MIC_90_ values (19). In general, oxazolidinone antibiotics maintained a high susceptibility to staphylococci and enterococci clinically isolated in China (19, 20). Nemonoxacin, a nonfluorinated quinolone, showed more excellent activity than fluoroquinolone comparators against the MSSA strains. *In vitro* activity is slightly greater than that of fluoroquinolones against MRSA (21). Oral nemonoxacin can achieve good clinical and microbiological efficacy in treating adult community-acquired pneumonia (CAP) caused by bacteria and atypical pathogens, which is not inferior to levofloxacin (22). Our research also showed that nemonoxacin was associated with higher susceptibility than levofloxacin in Staphylococcus spp. Additionally, nemonoxacin (MIC_50/90_, 0.25/4 mg/L) had lower MIC values for E. faecalis than did levofloxacin (MIC_50/90_, 2/32 mg/L). But there appeared in response to be a bimodal distribution of E. faecalis to nemonoxacin. A significant portion appeared at 0.25 mg/L, but a small subpopulation appeared between 2 and 4 mg/mL (Fig. 2c). This phenomenon may be related to bacterial resistance to levofloxacin. We found that nemonoxacin remained highly active against levofloxacin-susceptible E. faecalis (*n* = 388; MIC_50_, 0.25 mg/L; MIC_90_, 0.25 mg/L; MIC ranges, 0.015 to 1 mg/L), while it had lower activity against levofloxacin-resistant E. faecalis (*n* = 179; MIC_50_, 2 mg/L; MIC_90_, 4 mg/L; MIC ranges, 0.015 to 64 mg/L). The study by Adam et al. (23) also demonstrated the same phenomenon. A significant portion appeared at 0.125 mg/L, but a small subpopulation appeared at 1 mg/mL. And nemonoxacin was not so active against E. faecium *in vitro*. These results were consistent with reports from Canada and China (23, 24). Norvancomycin has a structure similar to that of vancomycin and has been commercially developed in China since 1967 (25). This study showed that norvancomycin had MIC values for most strains that were equal to or lower than those of vancomycin (Fig. 3 and 4) .

There are two limitations to this study. First, the strains used in this study were isolated 3 years ago, not in the last 1 to 2 years. Unfortunately, due to the global pandemic of COVID-19, the speed of collection and MIC determination of the 2020 and 2021 strains has been delayed. Although the data in this study cannot represent the susceptibility of the latest isolated strains, this study is one of the few multicenter studies in China, and its results have some reference value. Second, since neither the CLSI nor EUCAST has established clinical breakpoints for nemonoxacin and norvancomycin, the criteria for determining the susceptibility of nemonoxacin and norvancomycin in this study could only use the epidemiological cutoff values developed by China to determine the susceptibility of Staphylococcus spp. and *Enterococcus* spp. initially.

In conclusion, our study demonstrated that both eravacycline and tedizolid were highly active against clinical isolates of Staphylococcus spp. and *Enterococcus* spp. recently collected across China. Nemonoxacin showed potent *in vitro* activity against Staphylococcus spp. and E. faecalis but limited activity against E. faecium. Norvancomycin and ceftaroline displayed highly potent activity against Staphylococcus spp.

## MATERIALS AND METHODS

### Clinical isolates.

A total of 2,939 nonduplicate isolates of Gram-positive cocci were consecutively collected from 46 medical centers in 28 provinces or cities all over China in 2019, including Staphylococcus aureus (*n* = 1,631), Staphylococcus epidermidis (*n* = 121), Staphylococcus hominis (*n* = 61), Staphylococcus haemolyticus (*n* = 58), Enterococcus faecalis (*n* = 567), and Enterococcus faecium (*n* = 501). Species identification was performed at each participating site and was confirmed by the central laboratory using a Vitek-2 compact system (bioMérieux, Hazelwood, MO) or matrix-assisted laser desorption ionization–time of flight mass spectrometry (MALDI-TOF MS; Bruker, Billerica, MA). S. aureus ATCC 29213, S. aureus ATCC 25923, and E. faecalis ATCC 29212 were applied as the quality control strains for the antimicrobial susceptibility testing.

### Antimicrobial susceptibility testing.

MICs were determined by the broth microdilution method recommended by the Clinical and Laboratory Standards Institute (CLSI) (26). The MIC was defined as the lowest concentration of an antimicrobial agent that will inhibit the visible growth of a microorganism after overnight incubation. Testing was done as follows. (i) Antimicrobial agent solubilization and dilution were done according to the CLSI. Eravacycline, tigecycline, tedizolid, linezolid, norvancomycin, vancomycin, nemonoxacin, levofloxacin, ceftaroline, penicillin, erythromycin, clindamycin, gentamicin, trimethoprim-sulfamethoxazole, and nitrofurantoin were tested in our study. (ii) The final inoculum was 10^5^ CFU/mL. (iii) Cation-adjusted Mueller-Hinton broth (CAMHB; Becton) was used in this study. (iv) Ambient incubation of strains was performed at 35°C ± 2°C for 16 to 20 h (24 h for vancomycin and norvancomycin). (v) Quality control and interpretation of the results were performed according to 2021 CLSI breakpoints for all agents except for eravacycline, tigecycline, norvancomycin, and nemonoxacin, for which CLSI criteria are not available. Eravacycline and tigecycline MICs were interpreted using U.S. FDA and EUCAST MIC breakpoints. Norvancomycin and nemonoxacin (https://www.chinets.com/ECV) MICs were interpreted using the epidemiological cutoff (ECOFF) (27). Each MIC value was determined once.

### Statistical analysis.

Statistical indicators in this study, including MIC_50_, MIC_90_, MIC range, drug resistance rate, and susceptibility rate, were analyzed using WHONET software.

### Compliance with ethical standards.

This study protocol was approved by the Institutional Review Board of Huashan Hospital, Fudan University (no. 2019-460).

## References

[1] Browne AJ, Chipeta MG, Haines-Woodhouse G, Kumaran EPA, Hamadani BHK, Zaraa S, Henry NJ, Deshpande A, Reiner RC, Day NPJ, Lopez AD, Dunachie S, Moore CE, Stergachis A, Hay SI, Dolecek C. 2021. Global antibiotic consumption and usage in humans, 2000–18: a spatial modelling study. Lancet Planet Health 5:e893–e904. doi:10.1016/S2542-5196(21)00280-1. 34774223 PMC8654683

[2] O’Neill J. 2014. Antimicrobial resistance: tackling a crisis for the health and wealth of nations. Wellcome Trust, London, United Kingdom.

[3] Chin CY, Tipton KA, Farokhyfar M, Burd EM, Weiss DS, Rather PN. 2018. A high-frequency phenotypic switch links bacterial virulence and environmental survival in Acinetobacter baumannii. Nat Microbiol 3:563–569. doi:10.1038/s41564-018-0151-5. 29693659 PMC5921939

[4] Kmietowicz Z. 2017. Few novel antibiotics in the pipeline, WHO warns. BMJ 358:j4339. doi:10.1136/bmj.j4339. 28928155

[5] Sutradhar I, Ching C, Desai D, Suprenant M, Briars E, Heins Z, Khalil AS, Zaman MH. 2021. Computational model to quantify the growth of antibiotic-resistant bacteria in wastewater. mSystems 6:e00360-21. doi:10.1128/mSystems.00360-21. 34100640 PMC8579810

[6] Tacconelli E, Carrara E, Savoldi A, Harbarth S, Mendelson M, Monnet DL, Pulcini C, Kahlmeter G, Kluytmans J, Carmeli Y, Ouellette M, Outterson K, Patel J, Cavaleri M, Cox EM, Houchens CR, Grayson ML, Hansen P, Singh N, Theuretzbacher U, Magrini N, WHO Pathogens Priority List Working Group. 2018. Discovery, research, and development of new antibiotics: the WHO priority list of antibiotic-resistant bacteria and tuberculosis. Lancet Infect Dis 18:318–327. doi:10.1016/S1473-3099(17)30753-3. 29276051

[7] Pendleton JN, Gorman SP, Gilmore BF. 2013. Clinical relevance of the ESKAPE pathogens. Expert Rev Anti Infect Ther 11:297–308. doi:10.1586/eri.13.12. 23458769

[8] Lowy FD. 1998. Staphylococcus aureus infections. N Engl J Med 339:520–532. doi:10.1056/NEJM199808203390806. 9709046

[9] Lee AS, de Lencastre H, Garau J, Kluytmans J, Malhotra-Kumar S, Peschel A, Harbarth S. 2018. Methicillin-resistant Staphylococcus aureus. Nat Rev Dis Primers 4:18033. doi:10.1038/nrdp.2018.33. 29849094

[10] Fiore E, Van Tyne D, Gilmore MS. 2019. Pathogenicity of enterococci. Microbiol Spectr 7:7.4.9. doi:10.1128/microbiolspec.GPP3-0053-2018. PMC6629438 31298205

[11] Gagliotti C, Högberg LD, Billström H, Eckmanns T, Giske CG, Heuer OE, Jarlier V, Kahlmeter G, Lo Fo Wong D, Monen J, Murchan S, Simonsen GS, Šubelj M, Andrašević AT, Żabicka D, Žemličková H, Monnet DL, EARS-Net study group participants. 2021. Staphylococcus aureus bloodstream infections: diverging trends of meticillin-resistant and meticillin-susceptible isolates, EU/EEA, 2005 to 2018. Euro Surveill 26:2002094. doi:10.2807/1560-7917.ES.2021.26.46.2002094. 34794536 PMC8603406

[12] Brinkwirth S, Ayobami O, Eckmanns T, Markwart R. 2021. Hospital-acquired infections caused by enterococci: a systematic review and meta-analysis, WHO European Region, 1 January 2010 to 4 February 2020. Euro Surveill 26:2001628. doi:10.2807/1560-7917.ES.2021.26.45.2001628. 34763754 PMC8646982

[13] Markwart R, Willrich N, Haller S, Noll I, Koppe U, Werner G, Eckmanns T, Reuss A. 2019. The rise in vancomycin-resistant Enterococcus faecium in Germany: data from the German Antimicrobial Resistance Surveillance (ARS). Antimicrob Resist Infect Control 8:147. doi:10.1186/s13756-019-0594-3. 31485325 PMC6712849

[14] Trautmannsberger I, Kolberg L, Meyer-Buehn M, Huebner J, Werner G, Weber R, Heselich V, Schroepf S, Muench H-G, von Both U. 2022. Epidemiological and genetic characteristics of vancomycin-resistant Enterococcus faecium isolates in a university children’s hospital in Germany: 2019 to 2020. Antimicrob Resist Infect Control 11:48. doi:10.1186/s13756-022-01081-3. 35279207 PMC8917738

[15] Lee YR, Burton CE. 2019. Eravacycline, a newly approved fluorocycline. Eur J Clin Microbiol Infect Dis 38:1787–1794. doi:10.1007/s10096-019-03590-3. 31175478

[16] Morrissey I, Hawser S, Lob SH, Karlowsky JA, Bassetti M, Corey GR, Olesky M, Newman J, Fyfe C. 2020. In vitro activity of eravacycline against Gram-positive bacteria isolated in clinical laboratories worldwide from 2013 to 2017. Antimicrob Agents Chemother 64:e01715-19. doi:10.1128/AAC.01715-19. 31843997 PMC7038300

[17] Roger C, Roberts JA, Muller L. 2018. Clinical pharmacokinetics and pharmacodynamics of oxazolidinones. Clin Pharmacokinet 57:559–575. doi:10.1007/s40262-017-0601-x. 29063519

[18] Zhao C, Wang X, Zhang Y, Wang R, Wang Q, Li H, Wang H. 2019. In vitro activities of eravacycline against 336 isolates collected from 2012 to 2016 from 11 teaching hospitals in China. BMC Infect Dis 19:508. doi:10.1186/s12879-019-4093-1. 31182038 PMC6558774

[19] Guo Y, Yang Y, Zheng Y, Wu S, Yin D, Zhu D, Hu F. 2020. Comparative in vitro activities of ceftaroline and tedizolid against clinical strains of Staphylococcus aureus and Enterococcus: results from the China Antimicrobial Surveillance Network (CHINET) in 2018. Antimicrob Agents Chemother 64:e01461-20. doi:10.1128/AAC.01461-20. 32816731 PMC7577170

[20] Hu F, Guo Y, Yang Y, Zheng Y, Wu S, Jiang X, Zhu D, Wang F, China Antimicrobial Surveillance Network (CHINET) Study Group. 2019. Resistance reported from China Antimicrobial Surveillance Network (CHINET) in 2018. Eur J Clin Microbiol Infect Dis 38:2275–2281. doi:10.1007/s10096-019-03673-1. 31478103

[21] Watkins RR, Holubar M, David MZ. 2019. Antimicrobial resistance in methicillin-resistant Staphylococcus aureus to newer antimicrobial agents. Antimicrob Agents Chemother 63:e01216-19. doi:10.1128/AAC.01216-19. 31527033 PMC6879266

[22] Yuan J, Mo B, Ma Z, Lv Y, Cheng S-L, Yang Y, Tong Z, Wu R, Sun S, Cao Z, Wu J, Zhu D, Chang L, Zhang Y, Investigator Group of the Phase 3 Study on Oral Nemonoxacin. 2019. Safety and efficacy of oral nemonoxacin versus levofloxacin in treatment of community-acquired pneumonia: a phase 3, multicenter, randomized, double-blind, double-dummy, active-controlled, non-inferiority trial. J Microbiol Immunol Infect 52:35–44. doi:10.1016/j.jmii.2017.07.011. 30181096

[23] Adam HJ, Laing NM, King CR, Lulashnyk B, Hoban DJ, Zhanel GG. 2009. In vitro activity of nemonoxacin, a novel nonfluorinated quinolone, against 2,440 clinical isolates. Antimicrob Agents Chemother 53:4915–4920. doi:10.1128/AAC.00078-09. 19738018 PMC2772340

[24] Qin X, Huang H. 2014. Review of nemonoxacin with special focus on clinical development. Drug Des Devel Ther 8:765–774. 10.2147/DDDT.S63581 PMC4094567 25045247

[25] Jean SS, Chang LW, Hsueh PR. 2020. Tentative clinical breakpoints and epidemiological cut-off values of nemonoxacin for Streptococcus pneumoniae and Staphylococcus aureus isolates associated with community-acquired pneumonia. J Glob Antimicrob Resist 23:388–393. doi:10.1016/j.jgar.2020.10.017. 33207229

[26] Clinical and Laboratory Standards Institute. 2021. Performance standards for antimicrobial susceptibility testing, M100, 31th ed. Clinical and Laboratory Standards Institute, Wayne, PA.

[27] Yang Q, Li X, Jia P, Giske C, Kahlmeter G, Turnidge J, Yu Y, Lv Y, Wang M, Sun Z, Lin J, Li Y, Zheng B, Hu F, Guo Y, Chen Z, Li H, Zhang G, Zhang J, Kang W, Duan S, Wang T, Jing R, Xu Y, Chinese Committee on Antimicrobial Susceptibility Testing (ChiCAST). 2021. Determination of norvancomycin epidemiological cut-off values (ECOFFs) for Staphylococcus aureus, Staphylococcus epidermidis, Staphylococcus haemolyticus and Staphylococcus hominis. J Antimicrob Chemother 76:152–159. doi:10.1093/jac/dkaa414. 33057728

